# Cancer cell’s neuroendocrine feature can be acquired through cell-cell fusion during cancer-neural stem cell interaction

**DOI:** 10.1038/s41598-020-58118-z

**Published:** 2020-01-27

**Authors:** Liyuan Yin, Peizhen Hu, Xianping Shi, Weiping Qian, Haiyen E. Zhau, Stephen J. Pandol, Michael S. Lewis, Leland W. K. Chung, Ruoxiang Wang

**Affiliations:** 1 Lung Cancer Center, West China Hospital, Sichuan University, Chengdu, China; 2 Uro-Oncology Research, Department of Medicine, Cedars-Sinai Medical Center, Los Angeles, CA USA; 3 Department of Medicine, Cedars-Sinai Medical Center, Los Angeles, CA USA; 4 Department of Surgery, Emory University School of Medicine, Atlanta, GA USA; 5 Department of Pathology, Greater Los Angeles Veterans Affairs Health System, Los Angeles, CA USA

**Keywords:** Cancer microenvironment, Tumour heterogeneity

## Abstract

Advanced and therapy-resistant prostate tumors often display neural or neuroendocrine behavior. We assessed the consequences of prostate cancer cell interaction with neural cells, which are rich in the human prostate and resident of the prostate tumor. In 3-dimensional co-culture with neurospheres, red fluorescent human LNCaP cells formed agglomerates on the neurosphere surface. Upon induced neural differentiation, some red fluorescent cells showed morphology of fully differentiated neural cells, indicating fusion between the cancer and neural stem cells. These fusion hybrids survived for extended times in a quiescent state. A few eventually restarted cell division and propagated to form derivative hybrid progenies. Clones of the hybrid progenies were highly heterogeneous; most had lost prostatic and epithelial markers while some had acquired neural marker expression. These results indicate that cancer cells can fuse with bystander neural cells in the tumor microenvironment; and cancer cell fusion is a direct route to tumor cell heterogeneity.

## Introduction

Prostate cancer (PCa) has a multifaceted relationship with the nervous system. PCa progression is often accompanied by neurologic complications^1–3^ and loss of neurocognitive function^4,5^. PCa patients with neurologic events have poor quality of life, and patients with intracranial metastases have poor survival^6^. The nervous system seems tropistic to PCa progression, as neural peptides and hormones assist tumor growth and survival^7,8^. The peripheral nervous system may serve as a route for cancer infiltration, since PCa cells have high affinity to neural cells^9^ and perineuronal spaces are a thoroughfare for spreading tumor cells^10^.

Originating from the epithelial layer of the glandular prostate, PCa cells in clinical progression may acquire neural, endocrine, or neuroendocrine properties^11–13^. Neuroendocrinal PCa cells by themselves can secrete neural peptides and hormones promoting growth and survival in the absence of androgen, a mechanism of androgen-independent progression^14,15^. The focal or clustered distribution of neuroendocrine PCa cells in clinical specimens suggests clonal origin^16,17^. Neuroendocrine features in PCa are interpreted to result from transdifferentiation due to lineage plasticity^18^ and stem cell properties^19^. Soluble factors in the tumor microenvironment may modulate transdifferentiation by receptor-mediated signal transduction^14^, while additional exogeneous conditions may modulate via epigenetic mechanisms^20^.

We have demonstrated that PCa progression and metastasis is driven by cancer cell interaction with bystander resident cells in the tumor microenvironment^21–23^. Bystander neuroendocrine cells^11,12^ and innervating autonomic nerves^7,24^ are constituents as well. Using 3-dimensional (3-D) co-culture and xenograft tumor models, we found that direct contact with cancer cells converted bystander cells to malignant cells with permanent genomic alterations^25–27^. Mechanistically, LNCaP and other human PCa cells were found to be fusogenic, capable of forming cancer-stromal fusion hybrids once placed in direct contact, leading to the formation of heterogeneous fusion hybrid progenies^28^.

In the present study, we hypothesized that, like the fusion with bystander stromal cells of the tumor microenvironment, PCa cells may fuse with neural cells upon direct contact. We assessed the consequences of interaction between PCa and neural cells, by placing LNCaP cells in direct contact with rat neural stem cells (NSCs) under 3-D spheroid co-culture conditions^15,27^. The culture condition was then changed to induce NSC differentiation, while the fate of the PCa cells in co-culture was tracked to assess the effects of interaction. Results revealed that PCa cells could fuse with NSCs. Upon neural differentiation, most cancer-neural hybrids perished but some survived to display phenotypic heterogeneity, some even acquiring neural cell marker expression. This study thus revealed a previously unrecognized aspect of cancer-neural cell interaction.

## Materials and Methods

Protocol for xenograft tumor formation was approved by the Emory University IACUC committee (#254–2008). All methods and protocols were performed in accordance with institutional guidelines of the Emory University and the Cedars-Sinai Medical Center. Materials, data and associated protocols will be made available without undue qualifications in material transfer agreements.

### Cell culture reagents

Cull culture grade glucose, putrescine, selenite, apo-transferrin, insulin, and bovine serum albumin (BSA, Faction V) were purchased from Sigma-Aldrich (St. Louis, MO). Heparin was purchased from Alfa Aeasar (Ward Hill, MA). Basic fibroblast growth factor (bFGF) was purchased from USBiological (Swampscott, MA). Epidermal growth factor (EGF) was purchased form BD Biosciences (San Jose, CA). Other cell cultures reagents were purchased from Life Technologies (Carlsbad, CA).

### PCa cell cultures

We reported the establishment of LNCaP^RFP^, the RL-1 clone of the LNCaP human PCa cells (RRID: CVCL_0395) expressing an AsRed2 red fluorescence protein, selected by G418 selection (300 µg/ml)^28,29^. C4–2 and C4–2B LNCaP derivative cell lines^23,30^ were tagged with the same protocol. These cells were maintained on regular 10-cm culture dishes (CytoOne, USA Scientific, Ocala, FL) in PCa Medium, which was T-medium^21^ (Formula LS0020056DJ, Life Technologies) containing 10% fetal bovine serum (FBS, Atlanta Biologicals, Flowery Branch, GA), penicillin (100 unites/ml) and streptomycin (100 μg/ml), in a humidified incubator at 37 °C in atmospheric air supplemented with 5% CO_2_.

### Primary neurosphere culture

NSCs were prepared from subventricular zone (SVZ) of E18 rat cortex/hippocampus (BrainBits, Springfield, IL). An SVZ tissue section was placed in 1 ml ice-cold Neurosphere Medium, which was phenol red-free DMEM/F12 medium containing glucose (33.3 mM), putrescine (40 μM), selenite (30 nM), apo-transferrin (10 μg/ml), heparin (2 μg/ml), insulin (10 μg/ml), BSA (100 μg/ml), bFGF (20 ng/ml), EGF (20 ng/ml), B-27 (1: 100 dilution), penicillin (100 unites/ml) and streptomycin (100 μg/ml). The tissue was disintegrated by pipetting through a pipet tip, and tissue debris was removed after gravity sedimentation for 2 minutes. After washing twice in the same medium, the preparation was cultured (3.2 × 10^4^/0.2 ml/cm^2^ culture area) in regular 10-cm culture dishes in Neurosphere Medium for 2 weeks. Fresh medium was added to replace half of the culture volume every 4 days. After 14 days, primary neurospheres formed in the culture were collected as passage 1 (p1) NSCs and kept cryogenically for later use. Cell number and viability were monitored with trypan blue staining on a TC10 cell counter (BioRad Laboratories, Hercules, CA).

### Induced neural differentiation

NSCs grown in Neurosphere Medium were washed twice in phosphate buffered saline (PBS), and were plated in low density (100 neurospheres/ml) on a regular 10-cm culture dish. To induce neural differentiation with the method of growth factor withdrawal, Neurosphere Medium devoid of bFGF and EGF was used. To induce differentiation with the method of FBS addition, Neurosphere Medium containing 10% FBS was used. The third method of neural differentiation was culturing neuropspheres in the PCa medium. When necessary, the culture was maintained for 16 weeks with weekly replenishment of half of the medium.

### 3-D co-culture

To co-culture with PCa cells, NSCs were cultured first in Neurosphere Medium for 14 days for neurosphere formation. LNCaP^RFP^ cells were detached by trypsin-EDTA treatment, washed twice in PBS, reconstituted in single cell suspension in fresh Neurosphere Medium, and added to the 14-day NSC spheroid culture, in which 2 × 10^4^ LNCaP^RFP^ cells were mixed with 2 × 10^6^ NSCs in 2 ml Neurosphere Medium in one well of a regular 6-well plate (CytoOne, USA Scientific). The co-culture was maintained for 4 weeks. Half of the culture medium was replenished weekly without disturbing co-cultured cells.

### Assay for cell proliferation in 3-D co-culture

Cells in co-culture were prepared in single-cell suspension with trypsin-EDTA treatment; and were counted with a TC10 counter based on cell size. LNCaP^RFP^ cells, which showed a diameter of 14 µm in suspension, were counted from the peak between 12 µm and 16 µm. Rat NSCs had a diameter of 10.5 µm, for which the peak between 8.5 µm and 12 µm was counted. Quadruple counts were obtained from each sample.

### Species-specific genome detection with polymerase chain reaction (PCR)

Cells in co-culture were subjected to G418 (600 µg/ml) selection for 4 weeks with weekly medium change. Surviving cells were pooled for genomic DNA isolation with a DNeasy Blood & Tissue kit (Qiagen, Germantown, MD). Species-specific primer pairs for each short tandem repeat (STR) locus were synthesized according to published sequences for humans^31^ and rats^32^. For each locus, 10 ng genomic DNA was used as template. The experimental setting for PCR amplification has been reported^27,33^. PCR products were documented after electrophoretic fractionation on a 2% agarose gel containing ethidium bromide (1 µg/ml).

### Western blotting

The western blotting protocol has been reported^28,33^. Primary antibodies used in the study included mouse monoclonal antibodies to androgen receptor (AR, BD Biosciences, San Jose, CA), E-cadherin (E-cad, Cell Signaling Technology, Danvers, MA), prostate specific membrane antigen (PSMA, Proteintech, Rosemont, IL), chromograinin A (CgA, Proteintech), β-actin (Canta Cruz Biotechnology, Dallas, TX), and rabbit polyclonal antibodies to synaptophysin (SYP, Proteintech). Horseradish peroxidase-conjugated and species- and isotype-specific secondary antibodies and the SuperSignal West Dura substrate were obtained from Thermo Fisher Scientific (Waltham, MA). Results were documented with an Odyssey Fc imaging system (LI-COR, Lincoln, NE).

### Determining responsiveness to androgen-induced production of prostate specific antigen (PSA)

Cell culture medium was subjected to enzyme-linked immunosorbent assay (ELISA) for PSA concentration with our reported protocol^28,33^. Briefly, cells at 70% confluence were first kept under androgen-starvation conditions for 48 hours; and were then treated with the synthetic androgen methyltrienolone (R1881, Perkin Elmer, Waltham, MA) for 24 hours, when the culture medium was sampled for PSA detection.

### Assessment of tumorigenic potential

The protocol of xenograft tumor formation has been reported^30^. In brief, to assess local tumor growth, 6-week-old male NCr^nu/nu^ mice (National Cancer Institute, Frederick, MD) were inoculated subcutaneously on both flanks (2 × 10^6^ cells in 100 µl PBS/site, n = 5). Tumor dimension was measured with a caliper biweekly after inoculation. Humane endpoint was set as tumor volume reached 1.5 cm^3^, or hemorrhagic tumor ulceration occurred.

### Fluorescence microscopy

The protocol for red fluorescence imaging was previously reported^29^. In this study, for comparison purposes all the red fluorescent images were taken with fixed settings: 8 seconds for imaging at 40 × magnification, 2 seconds for imaging at 100 × magnification, and 1 second for imaging at 200 × magnification. Photoshop CS4 (Adobe Systems, San Jose, CA) was used to overlap images and Layer Style Blending Option software was used to demonstrate localization of red florescence in cultured cells.

## Results

We used LNCaP^RFP^, a clone of red fluorescent LNCaP human PCa cells, because RFP-tagging could facilitate cell fate tracking in complex co-culture systems^29^. As cancer cells are fusogenic, actively fusing to cells in juxtaposition^28^, RFP-tagging could reveal special aspects of cell interaction. Primary rat NSCs were used for their markedly distinctive morphologies and distinguishable heterogenic backgrounds from human PCa cells^34^.

### NSCs and induced neural differentiation

**Figure 1:**
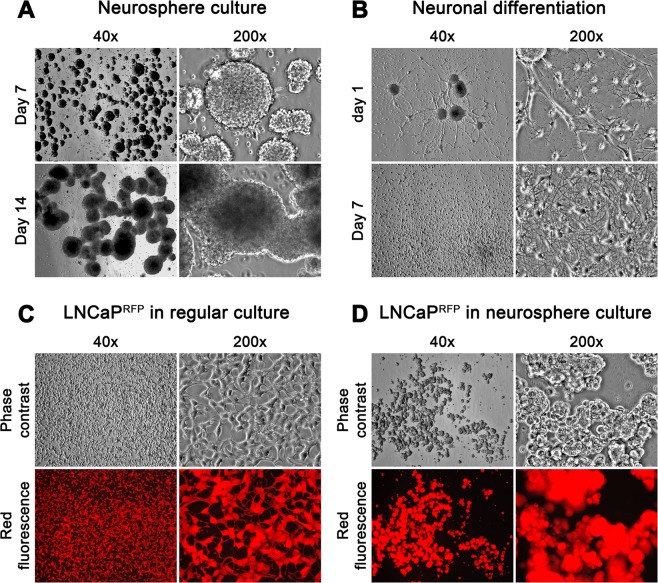
Participating cells of the 3-D co-culture system. Morphology and growth behavior for individual cell types are shown. (**A**) A subculture of rat NSCs in Neurosphere Medium is shown. Marked growth enlargement of the neurospheres was recorded from day 7 to day 14. (**B**) Once placed in PCa Medium, neurospheres became attached and started to differentiate with axon-like extensions appearing on day 1; and 3-D neurospheres differentiated into a 2-D neural network mesh in 7 days. (**C**) Attachment growth and epithelial morphology of LNCaP^RFP^ cells in 7-day PCa Medium culture. (**D**) The same number of LNCaP^RFP^ cells were seeded in Neurosphere Medium for 7 days. LNCaP^RFP^ adopted 3-D spheroid growth. These cells, placed back in PCa Medium, resumed attachment growth and epithelial morphology within 3 days without marked cell death (not shown).

We used an established protocol^35,36^ to expand NSCs of SVZ from E18 rat brain. These cells displayed slow but progressive proliferation in Neurosphere Medium, forming typical 3-D neuroprogenitor neurospheres in suspension in 2 weeks (Fig. 1A). With 6 specimens, we estimated an almost 200-fold expansion from 3.5 ± 0.817 × 10^6^ primary cells to 665 ± 136 × 10^6^ NSCs per SVZ specimen, with an NSC viability above 92% over the entire culture. Individual NSCs, disassociated from p1 neurospheres either by trituration or trypsinization, could be cultured in Neurosphere Medium into secondary neurospheres. No marked changes in morphology, growth rate or differentiation potential were observed in 4 consecutive passages. The primary neurosphere culture provided a convenient source of NSCs.

We evaluated stem cell properties in the expanded NSCs by inducing neural differentiation using 3 different methods. Using growth factor withdrawal^37^ or FBS addition^38^, we confirmed that the expanded NSCs underwent neural differentiation. We then modified the induction method by transferring neurospheres directly to the PCa Medium. In separate tests with 3 SVZ specimens, neurospheres responded to all 3 induction methods equally well. Attaching to the culture ware within 24 hours, neurospheres became flattened, with dendrite outgrowth appearing; followed by cells migrating out of the sphere concomitant to dendrite elongation into axon-like structures (Fig. 1B). Within 7 days, most neurosphere NSCs had migrated and differentiated into a 2-D neural network mesh. Upon differentiation induction, NSCs cease proliferation gradually after a few cell divisions^39^. Neural cells in the differentiated state could survive for at least 8 weeks in PCa Medium before the culture started to show cell loss, which became conspicuous at 12 weeks. The use of PCa Medium for inducing neural differentiation provided favorable cell culture system for investigating PCa-neural cell interaction.

Neurosphere Medium is rich in growth factors that promote organoid growth^40,41^. Human PCa cell lines adopt a spheroid growth in Neurosphere Medium as well. LNCaP^RFP^ cells, for instance, grew in an attached form in the PCa Medium (Fig. 1C) but would adopt spheroid growth in Neurosphere Medium (Fig. 1D), with a faster growth rate and enhanced PSA production. Compared to growth in PCa Medium, the growth rate of LNCaP^RFP^ cells increased almost a fold in 5 days (1.22 ± 0.10 × 10^6^ cells/well versus 2.21 ± 0.10 × 10^6^ cells/well in 6-well plates), while PSA production increased almost 46% in 24 hours (from 55.5 ± 6.20 ng/ml to 80.8 ± 2.14 ng/ml). The increased PSA production was most likely the effect of growth factors, which could promote LNCaP growth directly or through cross-talk with AR, the master transcription factor for prostate cell growth and PSA secretion^42^. Notably, after 2 weeks in Neurosphere Medium, the LNCaP^RFP^ spheroids transferred back to PCa Medium would regain their original attachment growth and morphology in 3 days, with no cell death occurring in the transition. Together with RFP tagging, these features served as baselines for tracking interaction with neural cells.

### PCa-NSC interaction in 3-D spheroid co-culture

**Figure 2:**
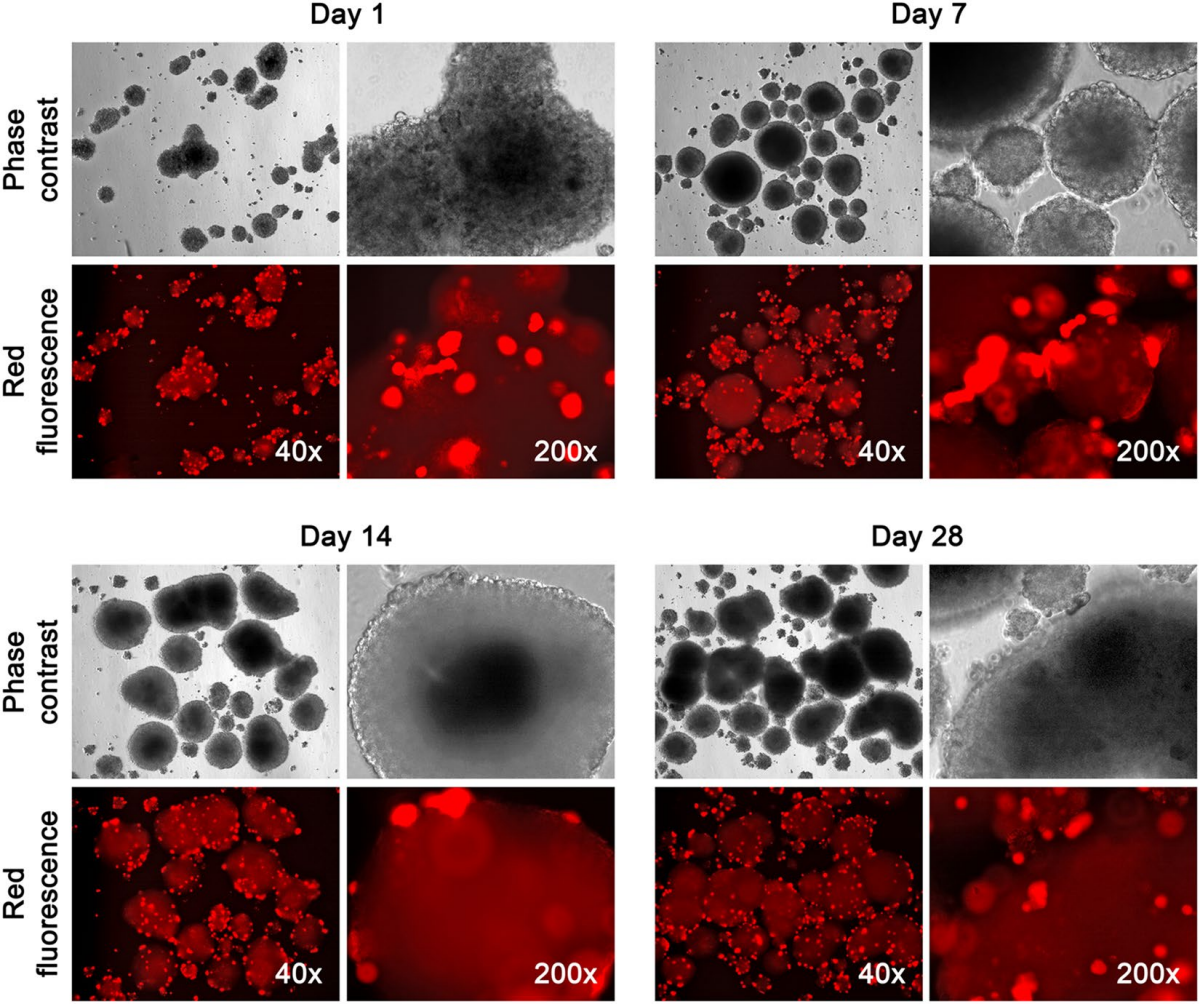
High affinity between LNCaP^RFP^ and NSCs. To assemble a 3-D co-culture, LNCaP^RFP^ cells in single cell suspension were added to a 14-day culture of NSC spheroids in Neurosphere Medium. LNCaP^RFP^ cells adhered to neurospheres, on which LNCaP^RFP^ survived and proliferated for the first two weeks, after which LNCaP^RFP^ growth rate declined and debris of red fluorescent cells was seen at day 28 of the co-culture.

We used neurospheres between passages 2 and 4 in 3-D co-culture with PCa cells. LNCaP^RFP^ cells in single-cell suspension were added directly to 14-day neurosphere cultures in Neurosphere Medium at an estimated 1: 100 cell ratio. Remarkably, all PCa cells attached to neurosphere surface, leaving virtually no free-floating red fluorescent cells 24 hours later (Fig. 2). LNCaP^RFP^ cells were highly affinitive to neurospheres.

Fluorescence microscopic inspection of 6 co-cultures for 4 weeks revealed several features of LNCaP^RFP^ cells. First, red fluorescent cells grew to become agglomerated on neurosphere surfaces, rarely infiltrating the neurosphere proper throughout the entire co-culture time (Fig. 2). Second, growth of LNCaP^RFP^ cells on neurosphere surfaces was discernibly slowed. Relative to monoculture growth rates either in PCa Medium (Fig. 1C) or Neurosphere Medium (Fig. 1D), LNCaP^RFP^ cells grew slower on neuosphere surfaces, in agreement with the antagonistic observation between PCa and peripheral innervation^43^. Third, there were 2 phases of LNCaP^RFP^ growth. In the first 2 weeks, slower but persistent growth resulted in an increase in red fluorescence. Further co-culture, however, showed gradual loss of red fluorescent cells and an increase in red fluorescent debris (Fig. 2). Cell number changes in a representative co-culture is summarized in Supplementary Figure S1. The cause of slowed growth and gradual death on neurosphere surfaces remains unclear.

### PCa-NSC fusion hybrids revealed by induced neural differentiation

To study the fate of co-cultured cells after 4 weeks, culture conditions were changed from Neurosphere Medium to PCa Medium to induce neural differentiation. The differentiated cells were maintained in PCa Medium for 12–16 weeks to assess the consequence of LNCaP^RFP^ and NSC interaction.

**Figure 3:**
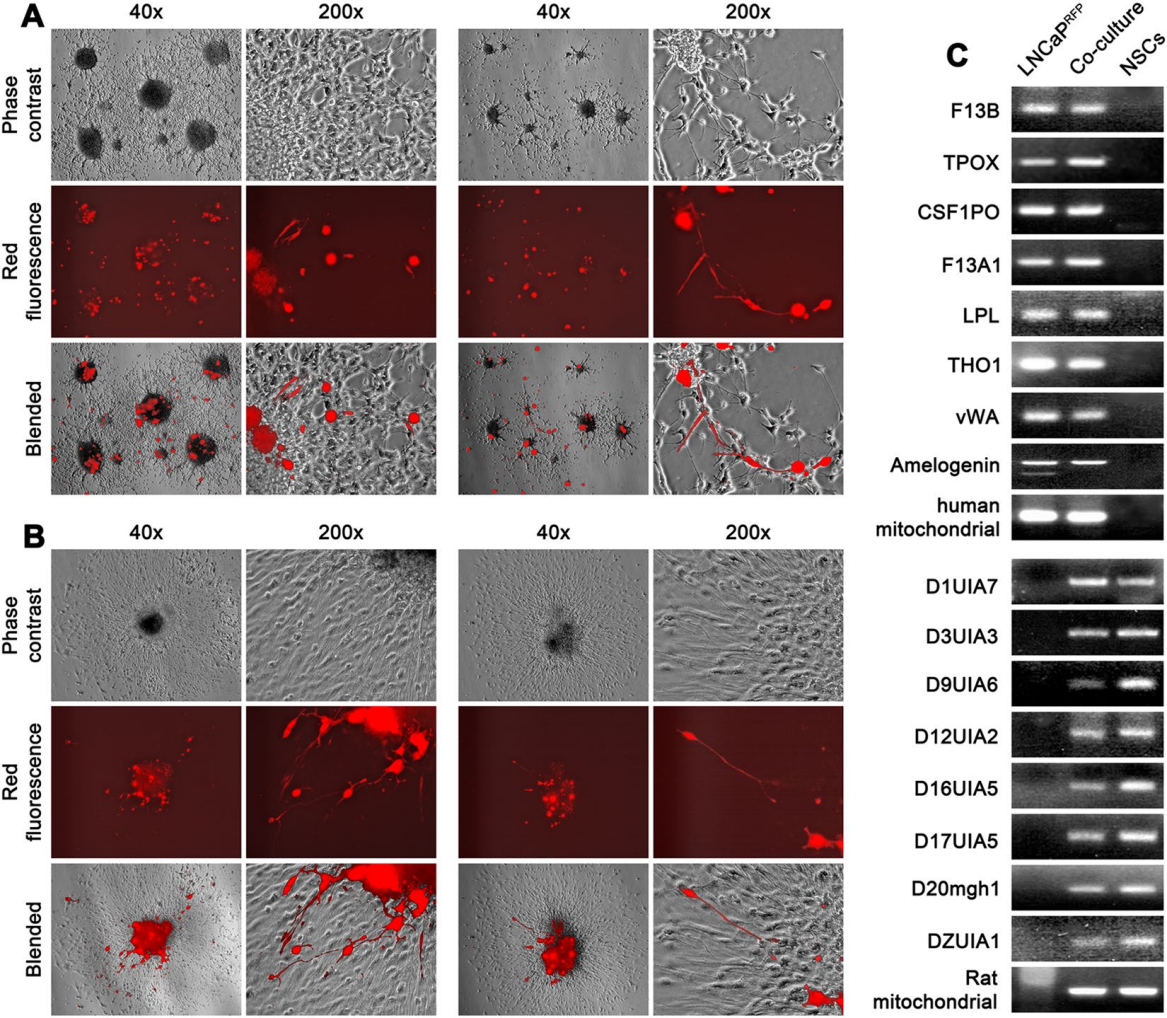
Marked death and discernible differentiation of LNCaP^RFP^ cells from 3-D co-culture. Induced differentiation in 3-D co-culture is shown in 4 representative view fields. (**A**) The first day of differentiation showed a large reduction in red fluorescent cell count. Left view field: most dead LNCaP^RFP^ cells were in the culture medium; much red fluorescent debris could still be seen in the differentiating cell monolayer. Right view field: Some surviving red fluorescent cells had neural cell morphology. (**B**) At day 7 of differentiation, red fluorescent cells with neural cell morphology could be frequently seen both around the center (left view field) or in the periphery (right view field) of neurosphere differentiation. For each view field, phase contrast and red fluorescence images are blended together (Blended) to facilitate localization of individual cells. (**C**) G418-selected red fluorescent cells with neural cell morphology were subjected to detection of human (the upper 9) and rat genomes (the lower 9) by PCR amplification of species-specific STR loci. Amplified DNA was detected by 2% agarose gel electrophoresis and ethidium bromide stain.

For LNCaP^RFP^ agglomerates on neurosphere surface, the primary response to induced neural differentiation was death of red fluorescent cells, resulting in large amount of fluorescent debris in suspension in the first 24 hours. When the debris was collected and re-plated in PCa Medium, few viable cells were recovered to form colonies. More than 90% of LNCaP^RFP^ cells in the agglomerates died in the first 24 hours of induced differentiation, leaving fewer red fluorescent cells in attachment to the differentiated neural network (Left view field, Fig. 3A). To quantitate cell number changes, we determined that a representative co-culture at 28 days contained 4.3 × 10^7^ NSCs and 9.4 × 10^4^ LNCaP^RFP^ cells. Seven days after induced neural differentiation, the culture contained 2.5 × 10^7^ differentiated NSCs but only 9.0 × 10^3^ LNCaP^RFP^ cells survived. Intriguingly, like the differentiated neural cells, more than 95% of the remaining red fluorescent cells were unable to enter the cell cycle and perished in the next 4 weeks. The death of LNCaP^RFP^ cells was attributable to differentiation-induced cell death, as we have investigated previously^19^.

For NSCs in the co-culture, the outstanding response was the presence of neural-cell shaped cells emitting red fluorescence (Right view field, Fig. 3A), which originated exclusively from LNCaP^RFP^ cells. These cells displayed various degrees of neural cell morphology, with elongated cell bodies and axon-like protrusions among the mesh of dendrites and axonal network, in the center (Left view field, Fig. 3B) or migrating to the periphery (Right view field, Fig. 3B) of neurospheres. Based on our previous studies^28^, these red fluorescent cells with neural cell morphology were fusion hybrids of parental LNCaP^RFP^ and NSCs. This was confirmed by detecting co-existent human and rat genomes in this cell type. Since LNCaP^RFP^ cells harbored an G418 selection marker, we treated the culture with a high dose of G418 (600 µg/ml) to remove rat cells not involved in cell fusion. Both human and rat genomic materials in the surviving cells were revealed by species-specific STR loci detection (Fig. 3C).

**Figure 4:**
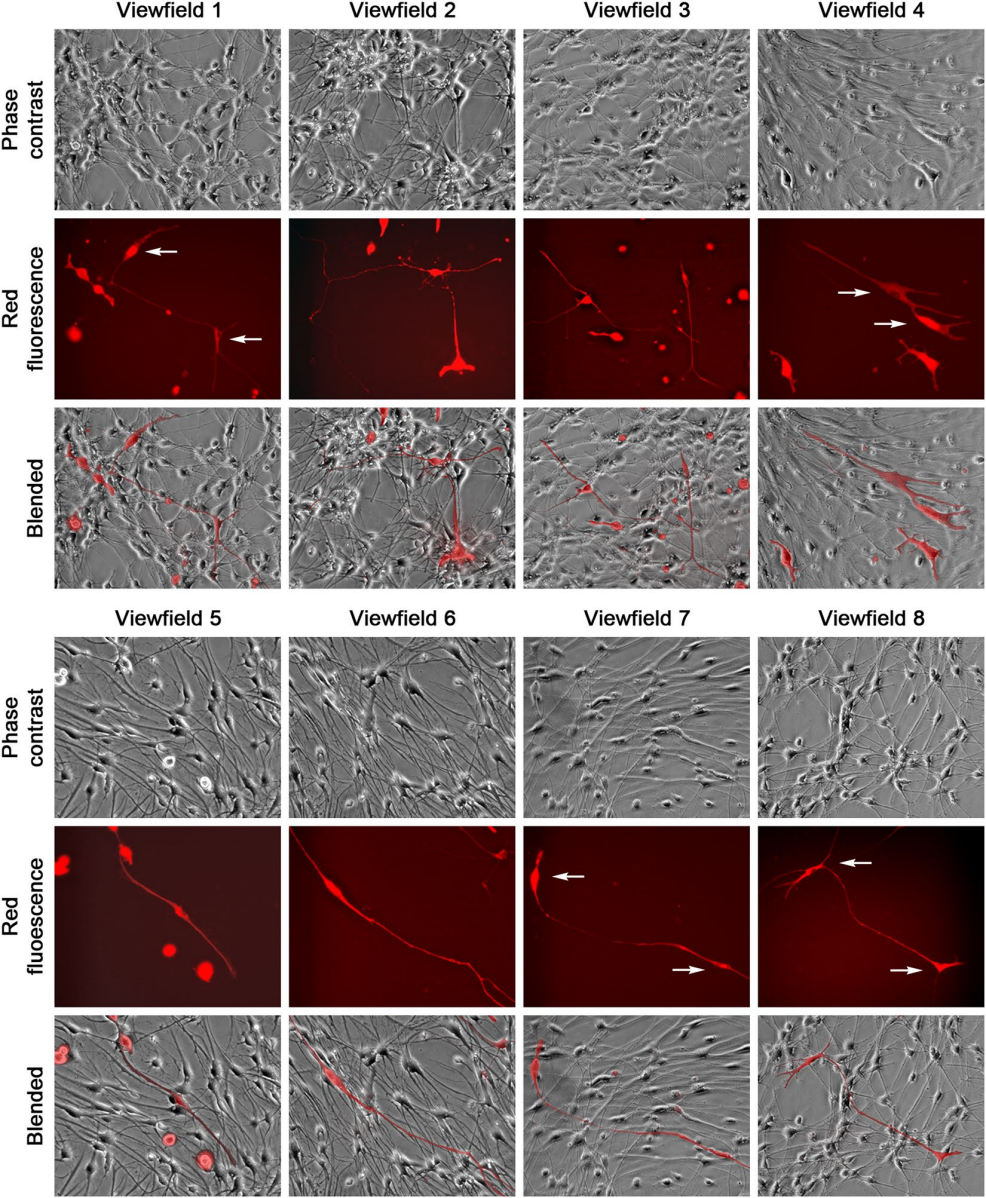
Fusion hybrids with neuron-like morphologies. Representative view fields at 200× magnification show the differentiation potential of the hybrid cells. The 8 view fields contain neuron-like fusion hybrids with unipolar, bipolar, or multipolar axon-like extensions. Arrow points to nucleus in fusion hybrid.

**Figure 5:**
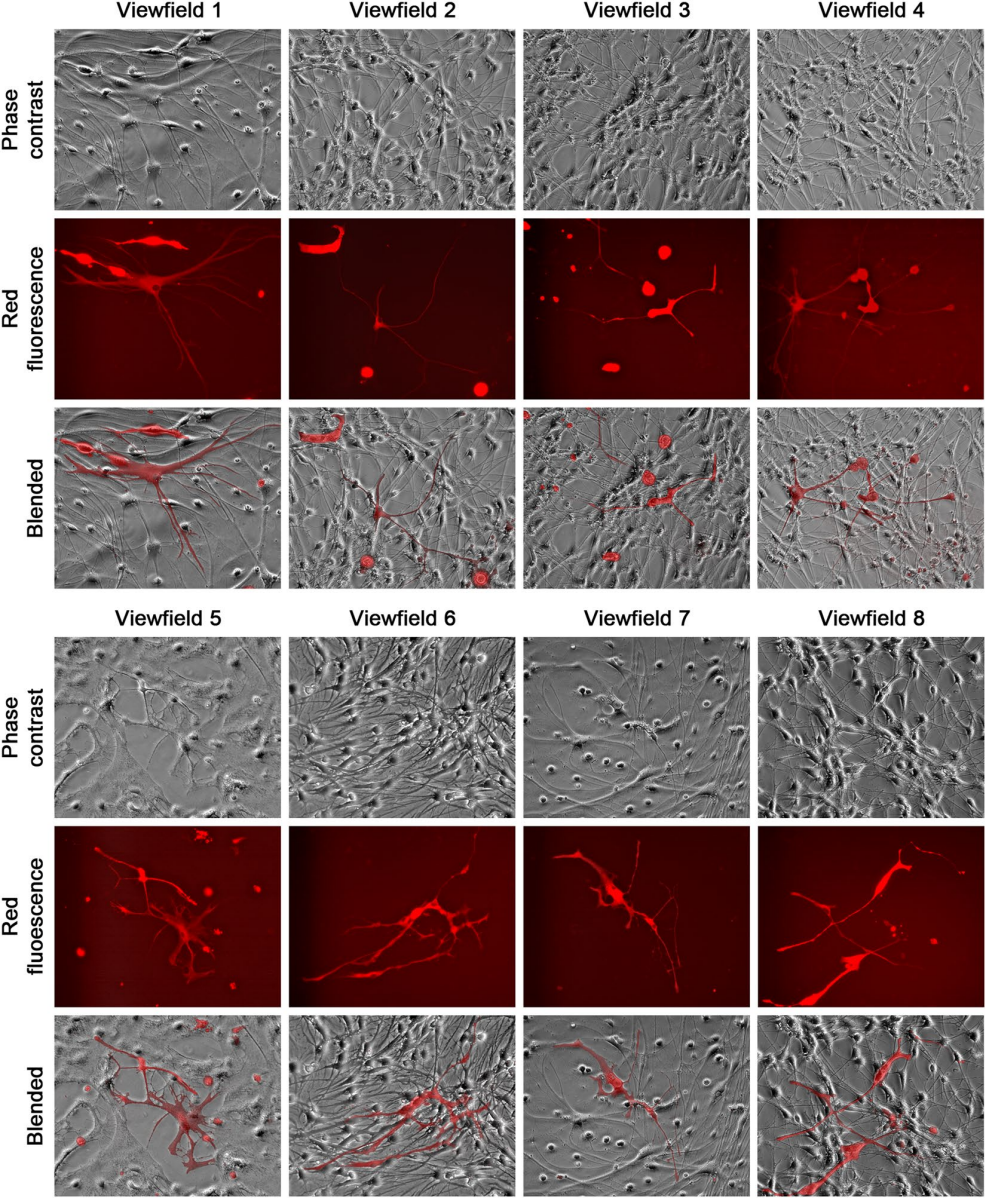
Fusion hybrids with astrocyte- or oligodendrocyte-like morphologies. Representative view fields at 200× magnification show astrocyte-like hybrids (view fields 1–4) and fusion hybrids with varied morphologies partially reminiscent of oligodendrocytes (view fields 5–8).

NSCs could differentiate into neurons, astrocytes and oligodendrocytes, each with distinct morphology in culture^37^. LNCaP^RFP^-NSC hybrids could be found displaying similar morphologies. Though an exact ratio could not be obtained due to the experimental setting, all three neural cell shapes were seen. Certain hybrid cells differentiated into neuron-like cells (Fig. 4) with multipolar (View fields 1–4), bipolar (View fields 5 and 6), or unipolar axon-like extensions (View fields 7 and 8). Relative to previous observations in PCa-stromal cell fusion^28^, the two nuclei in single hybrids were more difficult to visualize, mostly due to the smaller size of neural cells. The binuclear status, however, could be seen clearly in some hybrids (view fields 1, 4, 7 and 8). Hybrids in the astrocyte and oligodendrocyte lineages displayed more morphologic diversity (Fig. 5). While astrocyte-like hybrids were easy to identify (view fields 1–4), many other hybrids displayed varied morphologies reminiscent of oligodendrocytes (view fields 5–8). The fact that fusion hybrids inherited lineage features of the parental NSCs indicated that fusion took place between LNCaP^RFP^ and NSCs during 3-D co-culture. In comparison, we co-cultured LNCaP^RFP^ cells with neural network mesh in PCa Medium 2 weeks after induced neural differentiation. From 3 separate studies, little sign of red fluorescent neural cells was seen in 8 weeks of co-culture. Whether LNCaP^RFP^ cells have inherent preference for fusion with stem cells or the fusion depends on cell division remains to be investigated.

To estimate the frequency, low magnification (40×) fluorescence microscopy was used to count red fluorescent neural-cell-like cells among the mesh of differentiated neural cells in 24 random view fields 6 mm in diameter. In 3-D co-culture with 3 SVZ specimens, the number of hybrids among differentiated neural cells were estimated to be 164/64,450 (0.254%), 56/22,560 (0.248%), and 76/27,050 (0.281%), respectively. The 3-D co-culture protocol seemed to result in a consistent rate of PCa-neural cell fusion.

### Fate of the fusion hybrids

Cancer cell fusion with bystander neural cells of the tumor microenvironment would be highly relevant to cancer progression and metastasis, because fusion hybrids can generate progenies with tangible genotypic heterogeneity^28^. We tracked fusion hybrids from 6 separate 3-D co-cultures for 16 weeks post-differentiation to assess the fate of PCa-neural cell fusion. Based on these experiments, the fate of PCa-NSC hybrids was categorized into 3 destinies.

#### Growth arrest and death

**Figure 6:**
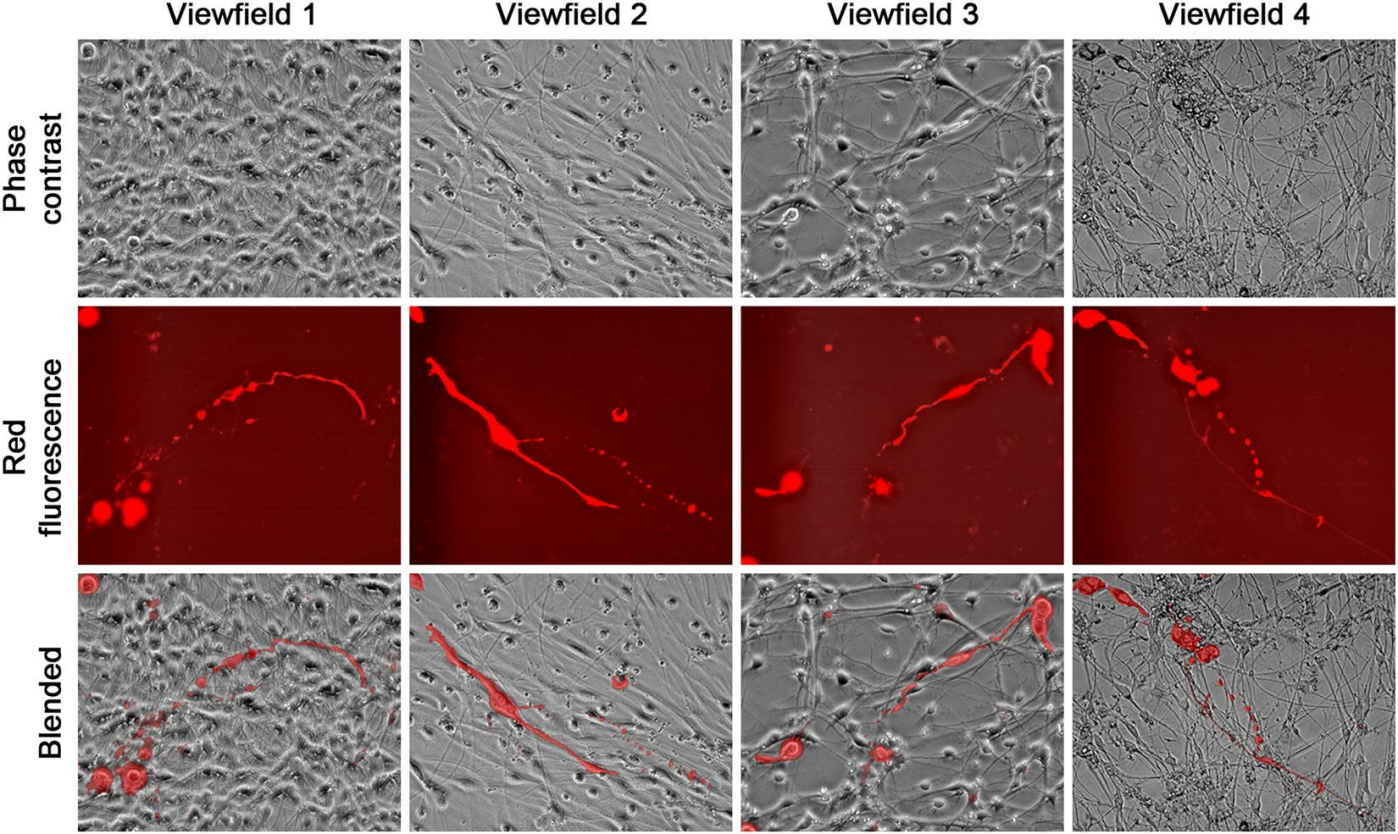
Death of hybrids. Representative view fields at 200× magnification show bead-like disintegration and debris disappears after changing the culture medium.

By tracking the existence of individual hybrids, we found that more than 95% of hybrid cells were in a state of growth arrest with varied life spans. About half of these cells died in the first 4 weeks of differentiation, with the long axon fragmenting into bead-like strings before disappearing (Fig. 6). Other cells survived much longer, up to 8 weeks. Comparing to the surrounding 2-D mesh of neural cells not involved in LNCaP^RFP^-NSC fusion, the hybrid neural cells seemed to have shortened lifespans.

#### Limited division and long survival

**Figure 7:**
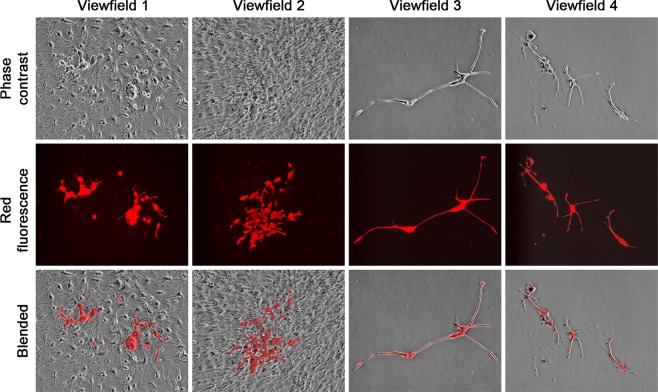
Failure in colony formation by fusion hybrid progenies. Representative view fields at 100× magnification are shown. From left, view fields 1 and 2: photos of clustered colony-like red fluorescent cells were taken 4 weeks after induced differentiation. Cells in these clusters survived a long time with no signs of proliferation at the end of the study (16 weeks). View fields 3 and 4: photos of cells in aborted cell division were taken at the end of the study.

After a quiescence of 4 to 8 weeks, a small fraction (<5%) of hybrids restarted to show signs of cell division, mostly cells with lesser neural but greater LNCaP cell morphology, shorter but wide and thicker in dimension (Fig. 7). These divisions, however, rarely led to full colony formation because division was limited (view fields 1 and 2), leaving a few progenies back to growth arrest. Some fusion hybrids with large cell sizes were seen stuck in a division process for many weeks until the end of the study (16 weeks) (view fields 3 and 4).

#### Hybrid progeny colony formation and appearance of clonal heterogeneity

**Figure 8:**
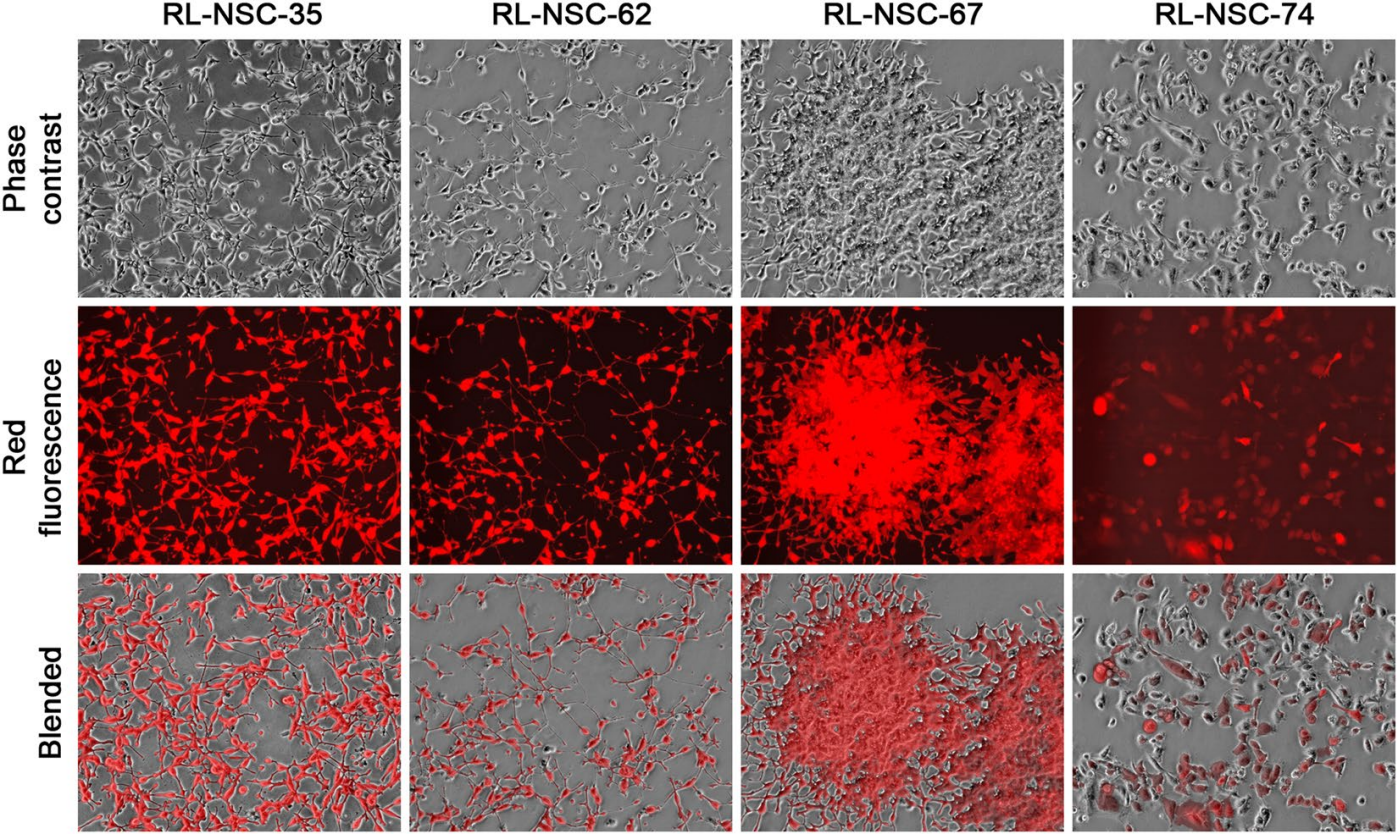
Successful survival and proliferation of fusion hybrid progenies. The morphology of 4 randomly selected sublines of the fusion hybrid progenies at passage 10 (100×). Compared to parental LNCaP^RFP^ or NSCs (Fig. 1), the different LR-NSC derivative sublines display distinct cell shapes and varied red fluorescence intensity, suggestive of acquired cellular heterogeneity.

Cell division in a few (<1%) of the hybrids became active and successful after weeks of quiescence, resulted in the formation of colonies that could be propagated beyond 10 continuous passages (Fig. 8). From the 6 co-cultures, we established 176 such derivative sublines (4, 21, 14, 26, 47, and 64 clones, respectively) as clones of red LNCaP and NSC hybrid progenies (RL-NSC-clones).

**Figure 9:**
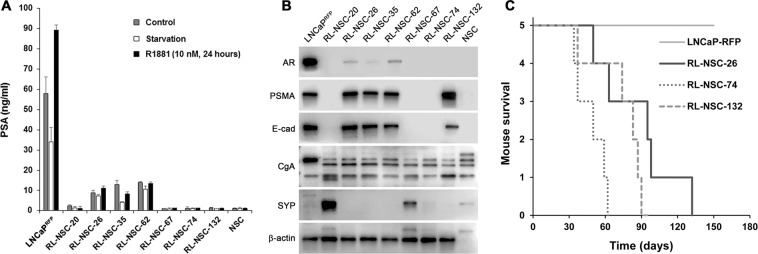
Acquired heterogeneity in hybrid progenies. Randomly selected RL-NSC clones were examined for changes in biomarker expression. (**A**) PSA ELISA results demonstrate that, compared to parental LNCaP^RFP^ cells, 4 of the 7 hybrid progeny clones lost PSA production (RL-NSC-20, −67, −74 and −132). The other 3 clones showed markedly inhibited PSA production (RL-NSC-26, −35, and −62). In addition, PSA production in these clones has become insensitive to androgen (R1881) stimulation. (**B**) Heterogeneous marker protein expression unveiled by western blotting analyses. Compared to LNCaP^RFP^ cells, all the 7 hybrid progeny clones became heterogeneous for AR, PSMA, and E-cad expression. As for neural markers, all hybrid progeny clones seemed to have acquired additional CgA expression, while clones of RL-NSC-20 and −67 have acquired the capability of expressing SYP markers. Each of the western blotting was repeated for at least twice and identical results were obtained. (**C**) Acquisition of tumorigenic potential was detected by xenograft tumor formation assay. Compared to LNCaP^RFP^ cells, which were not tumorigenic, all 3 hybrid progeny clones demonstrated varied capabilities of forming xenograft tumors.

The most salient features in these derivative clones were the high level of inter-clonal heterogeneities in cellular morphology, growth rate, and migration capacity. Though some clones displayed trace morphologies reminiscent of differentiated neural cells, a nearly equal number of clones showed no morphologic similarity with neural cells. A few clones were like LNCaP^RFP^ in morphology, while others had unique shapes. When cells from 7 randomly selected clones were examined for PSA production, diverse behaviors were found (Fig. 9A). Many hybrid progenies had lost the ability to produce PSA (RL-NSC-67, −74, and −132). Other clones retained PSA production, but the PSA production became much lowered and insensitive to androgen stimulation (RL-NSC-20, −26, −35, and −62). Marked loss of AR expression was revealed by western blotting (Fig. 9B), accompanied by altered expression of PSMA, a marker of glandular prostate luminal cells. There was also a loss of E-cad, a typical epithelial marker of parental LNCaP cells. Interestingly, the same cells showed altered patterns of neuroendocrine marker expression. Multiple products were detected in CgA blots, with an extra band suggesting an expression of CgA from parental rat neural cells. In addition, 2 of the 7 clones (RL-NSC-20 and −67) were detected with varied levels of SYP protein, rather like the parental rat neural cells. LNCaP^RFP^ cells are not tumorigenic in athymic mice with our inoculation protocol^30^. When 3 hybrid progeny clones were tested with the same protocol, however, rapid and progressive xenograft tumor formation was seen leading to euthanasia due to tumor burden and hemorrhagic ulceration (Fig. 9C). Comprehensive comparative examination of the hybrid progenies remains to be completed.

## Discussion

We used a 3-D co-culture system to simulate direct interaction between PCa and cells of the nervous system, which were resident bystander cells in the tumor microenvironment^7,9,24,43^. Primary rat NSCs were used as a reliable source of neural cells (Fig. 1) suitable for long-term observation before and after neural differentiation. LNCaP^RFP^ cells proliferated, albeit at a suppressed rate, on the surface of NSC spheroids (Fig. 2). Observations in this study suggested that fusion took place between LNCaP^RFP^ and NSCs during the 3-D co-culture (Fig. 3A). Aside from genomic validation (Fig. 3B) and morphological observation (Fig. 4), LNCaP^RFP^ seemingly fused with stem, progenitor or precursor cells in the neurosphere, as individual hybrids carried neuron (Fig. 4), astrocyte or oligodendrocyte morphologies (Fig. 5) revealed by induced neural differentiation. While many fusion hybrids died of growth arrest (Fig. 6), and many others failed in colony formation (Fig. 7), some hybrids with eventually succeeded in cell division, forming individual progeny populations with mutually heterogeneous behaviors (Fig. 8). The clonal heterogeneity became more pronounced at the molecular level, when the expression of prostate marker protein PSA was compared (Fig. 9A), in addition to divergent AR, PSMA, and E-cad levels and an altered neural marker CgA pattern (Fig. 9B). Interestingly, the 3 random clones tested have acquired xenograft tumor formation capabilities (Fig. 9C). We obtained similar results from 3-D co-culture of RFP-tagged C4–2 or C4–2B cells with the rat NSCs. Though the derivative clones have yet to be comprehensively characterized, this study unveils a natural history for the genesis of tumor cell heterogeneity, in which cell fusion is the initiating event.

Cell fusion is an essential biologic process^44–48^. As PCa cells are fusogenic^28^, spontaneity of the fusogeneity results in dynamic tumor cell heterogeneity^49^, the preponderant driver of cancer progression and metastasis. Fusion incidence rate was estimated to be around 0.25%, which we consider highly eventful, because the fusion could lead to the creation of hybrid progenies, with completely diverged genomic makeup and phenotypic behavior from their parental cancer cells. This study may also provide clues to understanding PCa acquisition of neuroendocrine features. Potential consequence of cancer cell fusion may impact every aspect of PCa progression and metastasis.

### Source of the neuroendocrine phenotype in PCa cells

How could PCa cells, somatic cells with an endodermal epithelium lineage origin, acquire phenotypic features of neuroendocrine cells of the neural crest of the ectoderm? The prevalent theory is neuroendocrine transdifferentiation in cancer cells, which may harbor lineage plasticity or stem cell properties, alternately expressing features of neural or endocrine cells via differentiation regulation. Our findings indicate that the neural behavior in this study results from fusion between PCa and neural cells, agreeable to previous discoveries^50,51^. Hybrid cells among a background of 2-D neural cell mesh (Figs. 3, 4 and 5) demonstrate the co-existence of PCa and neural cell features in singular cells. The pattern of neuroendocrine marker expression (*i.e*., CgA and SYP) in hybrid progenies suggests acquired neuroendocrine features (Fig. 9B). Fusion with NSCs is thus implicated as a cause of PCa cell acquisition of neural features. A detailed examination of the hybrid progeny clones is warranted.

### The mechanism of androgen-independent progression, tumor dormancy, and recurrence

Changes in AR expression and loss of androgen responsiveness are two common features of PCa progression^52–55^. In this study, all 7 randomly selected clones of fusion hybrids displayed null or suppressed responsiveness to androgen stimulation as gauged by R1881-stimulated PSA production (Fig. 9A). The responsiveness had association with loss of AR expression (Fig. 9B). It seems that individual clones of hybrid progenies were reprogrammed with distinctive gene expression patterns, and the reprogramming affected AR expression and androgen-response-related mechanisms.

Most metastatic brain tumors are insensitive to radiation or any other therapeutics^2,56,57^. Observations in our study suggest that fusion with NSCs provides an opportunity for PCa cells to be quiescent for an extended time. Hybrids in growth arrest would be inherently insensitive to conventional chemotherapeutics, which mainly target cell cycle mechanisms. A few hybrid progenies will eventually arise from quiescence with completely new cell entities. It will be intriguing to assess whether cell fusion leading to PCa dormancy is a factor in cancer recurrence.

### PCa-NSC fusion and neurocognitive function

Results of this study unveiled a previously unrecognized phenomenon: in addition to high affinity between the two cell types, PCa cells may fuse with neural cells. Though the 3-D spheroid co-culture made it difficult to appreciate specific harms inflicted on NSCs, we observed halted spheroid growth in later phases of the 3-D co-culture, suggesting a stressed state of the NSCs. Many neural cells involved in fusion had shortened survival (Fig. 6), and probably had compromised function. It is intriguing that over the long clinical course of PCa progression and metastasis, patient neural cells or neuroprogenitors are at chronic risk of fusion with cancer cells, as gradual loss of neural cells could eventually affect neurocognitive function.

### The cause of tumor cell heterogeneity

Cell fusion is a critical event in germ cell fertilization and somatic cell hybridization. Research on cancer cell fusion is accumulating convincing results showing that the mechanism of cell fusion can be hijacked for cancer cell survival, progression and metastasis^44^. The consequences and mechanism of cell fusion have been examined in the study of fertility and hybridoma formation^46,47^. Following cytoplastic fusion and somatic karyogamy, each heterokaryon undergoes extensive genomic re-organization to reduce the chromosome number^58^, probably using a yet-to-be-elucidated meiosis-like process of diploidization of polyploid genome^59^. In this regard, the inherent aneuploidy of the parental cancer cell, in combination with chromosomal mismatches in the hybrid, makes the meiosis-like process incomplete and instable, resulting in hybrid progenies with worsened aneuploidy and genomic instability. Cell fusion thus opens a window for cancer cells to be reprogrammed. It is not surprising that individual clones of the hybrid progenies showed diversified behaviors and distinctive marker protein expressions (Fig. 9). These results are in full agreement with our previous finding that cancer-stromal cell fusion was a cause of cancer cell heterogeneity^28^. Varied expression of marker proteins such as AR, PSA, E-cad, CgA and SYP are simply sign of somatic hybridization and reprogramming by individual fusion hybrids. Viewed even as a single cellular event, cancer cell fusion well explains all the histologic and pathophysiologic features of cancer.

## Supplementary information

Supplementary Figure S1.
