# Supplementary material for: Cancer cell’s neuroendocrine feature can be acquired through cell-cell fusion during cancer-neural stem cell interaction
Source: Sci Rep. 2020 Jan 27;10:1216. doi: 10.1038/s41598-020-58118-z (PMC6985266; doi:10.1038/s41598-020-58118-z)
Supplement: Supplementary file 1 — Supplementary Figure S1. [file 41598_2020_58118_MOESM1_ESM.pdf]

Cancer cell's neuroendocrine feature can be acquired through cell-cell fusion during cancer-neural stem cell interaction

Liyuan Yin[1,2], Peizhen Hu[2], Xianping Shi[3], Weiping Qian[4], Haiyen E. Zhau[2], Leland W. K. Chung[2], Michael S. Lewis[5], Stephen J. Pandol[3], and Ruoxiang Wang[2,5]

[1]Lung Cancer Center, West China Hospital, Sichuan University, Chengdu, China; [2]Uro-Oncology Research, [3]Department of Medicine, Cedars-Sinai Medical Center, Los Angeles, CA, USA; [5]Department of Pathology, Greater Los Angeles Veterans Affairs Health System, Los Angeles, CA, USA; and [4]Department of Surgery, Emory University School of Medicine, Atlanta, GA, USA.

Supplementary Data

Figure S1

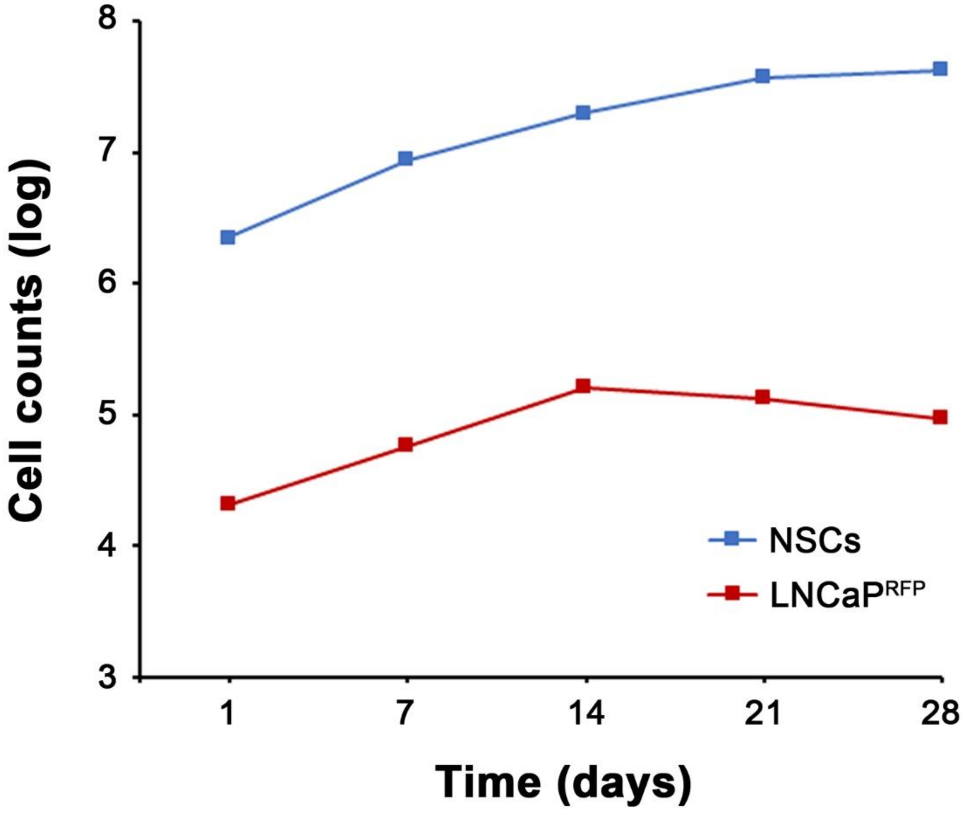

Figure S1. Cell number changes during co-culture. Cells in co-culture were prepared every 7 days in single-cell suspension with trypsin-EDTA treatment; and were counted with a TC10 counter based on cell size. Quadruple counts were obtained from each sample. The data are shown with the mean of quadruple counts. For all the data points, standard deviations are less than 5% of the mean and are not shown.
